# Effects of sEV derived from SHED and DPSC on the proliferation, migration and osteogenesis of PDLSC

**DOI:** 10.1016/j.reth.2023.09.009

**Published:** 2023-09-22

**Authors:** Yexin Zheng, Hui Lu, Qing Mu, Ping Yi, Ling Lin, Pei Li, Dongsheng Yu, Wei Zhao

**Affiliations:** Guanghua School of Stomatology, Hospital of Stomatology, Guangdong Provincial Key Laboratory of Stomatology, Sun Yat-Sen University, Guangzhou 510055, China

**Keywords:** Periodontitis, Small extracellular vesicle, Periodontal ligament stem cell, Proliferation, Migration, Osteogenesis

## Abstract

**Introduction:**

Periodontitis is a highly prevalent oral disease characterized by irreversible bone resorption and tooth loss. The proliferation, migration and osteogenic differentiation of periodontal ligament stem cell (PDLSC) are crucial to the regeneration of periodontal bone defects. There is increasing evidence that small extracellular vesicle (sEV) derived from pulp stem cell, including human exfoliated deciduous teeth stem cell (SHED) and human dental pulp stem cell (DPSC), is a potential mediator for bone tissue regeneration. However, which one is more suitable for periodontal bone formation still remains to be studied.

**Methods:**

In this study, NTA and BCA were performed to compare the productivity of sEV derived from SHED (SHED-sEV) and sEV derived from DPSC (DPSC-sEV). CCK-8, transwell assay, alkaline phosphatase staining and activity assay, alizarin red staining, qRT-PCR, and western blotting were conducted to detect the proliferation, migration, and osteogenesis of PDLSCs coculturing with SHED-sEV or DPSC-sEV.

**Results:**

The secretory efficiency of SHED-sEV was much higher than that of DPSC-sEV. The cellular uptake of sEVs could promote the proliferation, migration and osteogenesis of DPLSCs. Compared with DPSC-sEV, SHED-sEV showed better ability in such promotion.

**Conclusions:**

SHED-sEV showed higher productivity and better osteogenic induction ability than DPSC-sEV. Thus, SHED-sEV may be a more promising candidate for periodontal bone regeneration.

## 1 Introduction

Periodontitis is a highly prevalent oral disease characterized by irreversible bone resorption and tooth loss [1]. Currently, treatments for periodontal bone defects include guided bone regeneration (GBR), autologous bone transplantation, and synthetic materials combined with growth factors [2,3]. Nevertheless, these methods all have drawbacks. For example, the therapeutic effect of GBR is unpredictable because of the limitation of new-formed bone and the lack of local vascularization; autologous bone is difficult to acquire, and the donor bone area would be susceptible to infection; synthetic materials and autologous bone face the risk of immune rejection [4,5]. None of these methods can restore the original anatomical and physiological function of periodontal hard tissue. Complete and functional periodontal bone regeneration still remains a clinical challenge [6].

Osteogenic differentiation of PDLSCs is an important event in the repair of alveolar bone defects in periodontitis [7]. A large number of studies have shown that PDLSCs can be used to treat periodontal bone defects [8]. However, their source and osteogenesis ability are limited, and the safety of stem cell therapy largely restricts their clinical application [9]. In recent years, cell-free therapy based on recruitment of endogenous stem cells has received widespread attention. It depends on bioactive molecules that create appropriate local microenvironment and regulate cell behavior [10]. Compared to stem cell transplantation, this method is more clinically feasible as there is no limitation of cell source, less chance of infection and lower risk of immune rejection. Additionally, biological agents can be produced and stored at −20 °C for 6 months without loss of bioactivity. A lot of studies used growth factors (GF) as bioactive component in cell-free therapy, including platelet-rich fibrin and platelet-rich plasma [11,12]. However, the half-life of GFs is short in the extracellular space so they fail to conduct continuous regeneration effectively [13]. Therefore, there is an urgent need to develop a strategy to overcome current difficulties in regenerative medicine.

Extracellular vesicle (EV) is encapsulated particle secreted by all types of parental cells and exist in most biological fluids, such as blood, saliva, urine, breast milk, and cerebrospinal fluid [14,15]. Small extracellular vesicle (sEV) is the most popular type of extracellular vesicle (EV) with a diameter of about 30–150 nm and a density of 1.10–1.18 g/ml, also known as exosome [16,17]. SEV is an important medium for information exchange and material transportation between cells, carrying a variety of proteins, lipids and genetic materials, regulating physiological processes such as cell proliferation, migration and differentiation and taking part in tissue regeneration, immune response regulation and other activities [18,19]. Unlike cells, sEV can't replicate themselves after being injected into the body, so they have a lower risk of tumorigenesis and pathogen metastasis [20]. There is increasing evidence that sEV secreted by pulp stem cells, including human exfoliated deciduous teeth stem cell (SHED) and human dental pulp stem cell (DPSC), is a potential mediator for bone tissue regeneration [21]. However, which one is more suitable for periodontal bone formation is still unclear. Although SHEDs have stronger proliferation, migration and osteogenesis ability than DPSCs [[22], [23], [24]], there is not enough studies supporting that the biological effects of sEV completely correspond to their parent cells. The purpose of this study was to compare the productive efficiency of sEV secreted via SHED (SHED-sEV) and sEV secreted via DPSC (DPSC-sEV), and to compare the benefits of SHED-sEV and DPSC-sEV in promoting periodontal bone regeneration, providing theoretical basis for choosing a better source of sEV in periodontal tissue engineering.

## 2 Method

### 2.1 Isolation and characterization of cells

Briefly, caries-free deciduous teeth were collected from 6- to 10-year-old children, and healthy third molars were collected from 18- to 25-year-old adults. Ethical approval was obtained from the School of Stomatology, Sun Yat-sen University. The teeth were placed in Dulbecco's modified Eagle's medium (DMEM. Gibco, USA) with 2% penicillin/streptomycin (P/S. Gibco) and taken to the laboratory within 4 h of extraction.

The periodontal ligament was scraped from the middle third of the root surface of permanent teeth. Pulp tissue was extracted with a sterilized dental bur. The following steps of isolating and culturing SHEDs, DPSCs, and DPLSCs were the same. The harvested dental pulp/periodontal ligament were cut into pieces and digested with collagenase type I (3 mg/ml. Sigma-Aldrich, USA) and dispase (4 mg/ml. Roche, USA) at 37 °C for 30 min. Partially digested tissues were centrifuged at 1000 g for 4 min at room temperature. Tissue pellet was resuspended in regular medium, composed of Dulbecco's modified Eagle's medium: nutrient mixture F12 (DMEM/F12. Gibco), 20% fetal bovine serum (FBS. Gibco), and 2% P/S, then cultured in 37 °C and 5% CO_2_ incubator.

Morphology of primary cells (P0) and third-passage (P3) cells was observed by an inverted microscope (Zeiss, Oberkochen, Germany). SHEDs, DPSCs, and DPLSCs at passage 3 were subjected to flow cytometric analysis to detect the expression of cell surface markers for CD34, CD44, CD45, CD73, CD90, and CD105 (BD Pharmingen, San Diego, CA, USA). Flow cytometry was performed with a Beckman Coulter CytoFlex system (Beckman Coulter, Fullerton, CA, USA).

Cells were passaged when they reached 80–90% confluency. Fresh medium was added every 2–3 days and passage 3–5 cells were used for the in vitro experiments.

### 2.2 Isolation, characterization and quantification of sEV

DPSCs and SHEDs at passage 5 were seeded in 9-cm plates. Cells were rinsed with PBS and cultured in serum-free medium for 48 h. The conditioned medium was collected and centrifuged sequentially at 300×*g* for 10 min and 2000×*g* for another 15 min to eliminate cell debris. Then the sample was centrifuged at 10,000×*g* for 30 min to remove large vesicles, followed by ultracentrifuged at 100,000×*g* for 80 min (Beckman Coulter, Germany). The sEV pellet was resuspended and stored at −80 °C for subsequent use.

The sEV proteins were quantified by a Micro BCA Protein Assay Kit (CWBIO, China). The freshly extracted sEV was resuspended and diluted by pure water with a PH of 7 for nanoparticle tracking analysis (NTA). The particle size distribution, concentration, and zeta potential of sEV were identified using a ZetaView (Particle Metrix, German). The morphology of sEV was determined with a transmission electron microscope (TEM). The suspension was loaded onto a copper grid at room temperature for 20 min. After drying, the sample was negatively stained with 2% (w/v) phosphotungstic acid for 5 min. Micrographs were obtained under a transmission electron microscope (HITACHI H-7650, Japan). Furthermore, the sEV-specific protein markers CD63 and TSG101 were examined by western blotting.

### 2.3 SEV labeling and uptake

Dil (Zetalife, America) was used to label SHED-sEV and DPSC-sEV with red fluorescence according to the manufacturer's instructions. For investigation of whether PDLSCs could uptake the two types of sEVs, PDLSCs were seeded on a laser-scanning confocal dish at a concentration of 1 × 10^4^ cells/dish. After PDLSCs were attached, Dil-labeled SHED-sEV and DPSC-sEV (5 μg/ml) were added. Twenty-four hours of coculture later, PDLSCs were fixed with 4% paraformaldehyde, subsequently stained by 4′,6-diamidino-2-phenylindole (DAPI. Solarbio, China) and phalloidin (Macklin, China). The uptake of sEVs was visualized by confocal microscopy (ZEISS LSM 980, Germany).

### 2.4 Cell proliferation assay

PDLSCs were plated on 96-well plates at a concentration of 5 × 10^3^ cells/well. Different concentrations (0, 5, 10 and 15 μg/ml) of SHED-sEV or DPSC-sEV were added and supplied with culture medium. PDLSCs proliferation was tested by means of Cell Counting Kit-8 (CCK-8. DOJINDO, Japan). After the indicated time points for 1, 3, 5, and 7 days of incubation, the culture medium of each well was replaced with 10 μL of CCK-8 solution and 100 μL DMEM, after which the plates were incubated for 1 h at 37 °C. The OD value of each well was measured by means of a microplate reader (BioTek Epoch 2, USA) at a wave length of 450 nm.

### 2.5 Cell migration assay

PDLSCs were seeded into upper chamber of 8 μm pores transwell inserts (Corning, USA) at a density of 2 × 10^5^ cells/ml in 100 μL of FBS-free medium. Culture medium with different concentrations (0, 5, 10 and 15 μg/ml) of SHED-sEV or DPSC-sEV were contained in lower chambers. Cells on the upper side of the filter were carefully removed with a cotton swab after 24 h, and the filters were fixed in formaldehyde and then stained with 0.1% crystal violet solution. Migrated cells were photographed and counted with an inverted microscope (Axiovert 40. Zeiss, Germany).

### 2.6 Alkaline phosphatase staining and alkaline phosphatase activity assay

The osteogenic induction medium (OIM) is DMEM with 10% FBS, 1% P/S, 0.1 μM dexamethasone (Sigma-Aldrich), 10 Mm β-glycerophosphate (Sigma-Aldrich), and 50 μM ascorbic acid (Sigma-Aldrich). PDLSCs were seeded in six-well plates at a density of 2 × 10^5^ per well. They were incubated in OIM with or without SHED-derived sEV (15 μg/ml) or DPSC-derived sEV (15 μg/ml) for 7 days. For ALP activity determination, the ALP assay kit (Nanjing Jiancheng Bioengineering Institute, China) was applied after 7 days of coculture. And alkaline phosphatase staining (ALP) was performed using an NBT/BCIP staining kit (Beyotime, China). Cells were fixed in 4% paraformaldehyde for 30 min, and then ALP staining was performed according to the manufacturer's instructions.

### 2.7 Alizarin red staining

Alizarin red staining was conducted to assess mineralization. PDLSCs were seeded in six-well plates at a density of 2 × 10^5^ per well. They were incubated in OIM with or without SHED-derived sEV (15 μg/ml) or DPSC-derived sEV (15 μg/ml) for 14 days. Then the cells were fixed in 4% paraformaldehyde and stained with alizarin red (Cyagen Biosciences Inc., China) at room temperature. Finally, mineralized nodules were photographed with an inverted microscope (Axiovert 40. Zeiss, Germany). For mineral deposition quantification, the calcium deposits were dissolved with 10% cetylpyridinium chloride monohydrate. The OD value of the extracted stain was measured at a wave length of 562 nm in a 96-well plate.

### 2.8 Quantitative real-time reverse transcription polymerase chain-reaction (qRT-PCR)

PDLSCs were seeded in 6-well plates at a density of 2 × 10^5^ per well, then treated with SHED-sEV or DPSC-sEV for 14 days. Total RNA was extracted with RNA-quick Purification Kit (ESscience, China) following the manufacturer's instruction. RNA concentration was determined by light absorbance at 260 nm (NanoDrop 2000c, Thermo), and purity was assessed according to A260/A280 ratios. Complementary DNA was prepared through reverse transcription kit (Takara Bio Inc, Shiga, Japan). The expression of osteogenic related genes (ALP, RUNX2, COL1, and OCN) was quantified by qRT-PCR, which was performed on a LightCycler 480 Detection System (Roche, Basel, Switzerland) with an SYBR Green Kit (Roche) and gene-specific primers. The primers used in the reaction were itemized in Table 1. GAPDH was used as the internal control. The relative gene expression fold changes in each group were calculated and compared by the 2^−ΔΔCT^ method.

**Table 1:** Primer Sequence Used in qRT-PCR.

gene	primer	sequence
RUNX2	forward	5′-CCACTGAACCAAAAAGAAATCCC-3′
	reverse	5′-GAAAACAACACATAGCCAAACGC-3′
OCN	forward	5′-AGCAAAGGTGCAGCCTTTGT-3′
	reverse	5′-GCGCCTGGGTCTCTTCACT-3′
COL1	forward	5′-CGATGGATTCCAGTTCGAGTATG-3′
	reverse	5′-TGTTCTTGCAGTGGTAGGTGATG-3′
ALP	forward	5′-CCTCCTCGGAAGACACTCTG-3′
	reverse	5′-GCAGTGAAGGGCTTCTTGTC-3′
GAPDH	forward	5′-TCTCCTCTGACTTCAACAGCGACA-3′
	reverse	5′-CCCTGTTGCTGTAGCCAAATTCGT-3′

### 2.9 Western blotting

PDLSCs were seeded in 6-well plates at a density of 2 × 10^5^ per well, then treated with SHED-sEV or DPSC-sEV for 14 days. Briefly, the RIPA buffer (KeyGenBioTECH, China) containing 1 mmol/L protease inhibitor cocktail (CWBIO, China) was used to lyse cells. Protein concentration were measured by means of the BCA assay kit (CWBIO). A 15 μg quantity of protein was subjected to Tris-Glycine SDS-PAGE (CWBIO) and transferred onto polyvinylidene fluoride membranes (Millipore). Next, 5% fat-free milk in Tris-buffered saline with Tween (TBST. 10 mmol/L Tris-HCl, 50 mmol/L NaCl, and 0.25%Tween 20) was used to block membranes for 1 h at room temperature, then incubated with the primary antibodies against OCN (Abcam, USA), RUNX2 (Cell Signaling Technology, USA), COL1 (Abcam), ALP (Abcam), and GAPDH (Cell Signaling Technology) for 20 h at 4 °C. All of the antibodies were diluted 1:1000 as described in the manufacturer's protocol. Subsequently, after 1 h of incubation with secondary antibody (1:5000. Cell Signaling Technology), bands were detected with an enhanced chemiluminescence detection system (Millipore) and quantified using ImageJ software.

### 2.10 Statistical analysis

All data were expressed as the mean ± standard deviation of three independently repeated experiments. Statistical significance for the two groups was assessed using Student's t-test. Statistics were carried out using the Prism GraphPad 6 software. Statistically significance was recognized at p < 0.05.

## 3 Results

### 3.1 Characterization of SHEDs, DPSCs, and DPLSCs

**Fig. 1:**
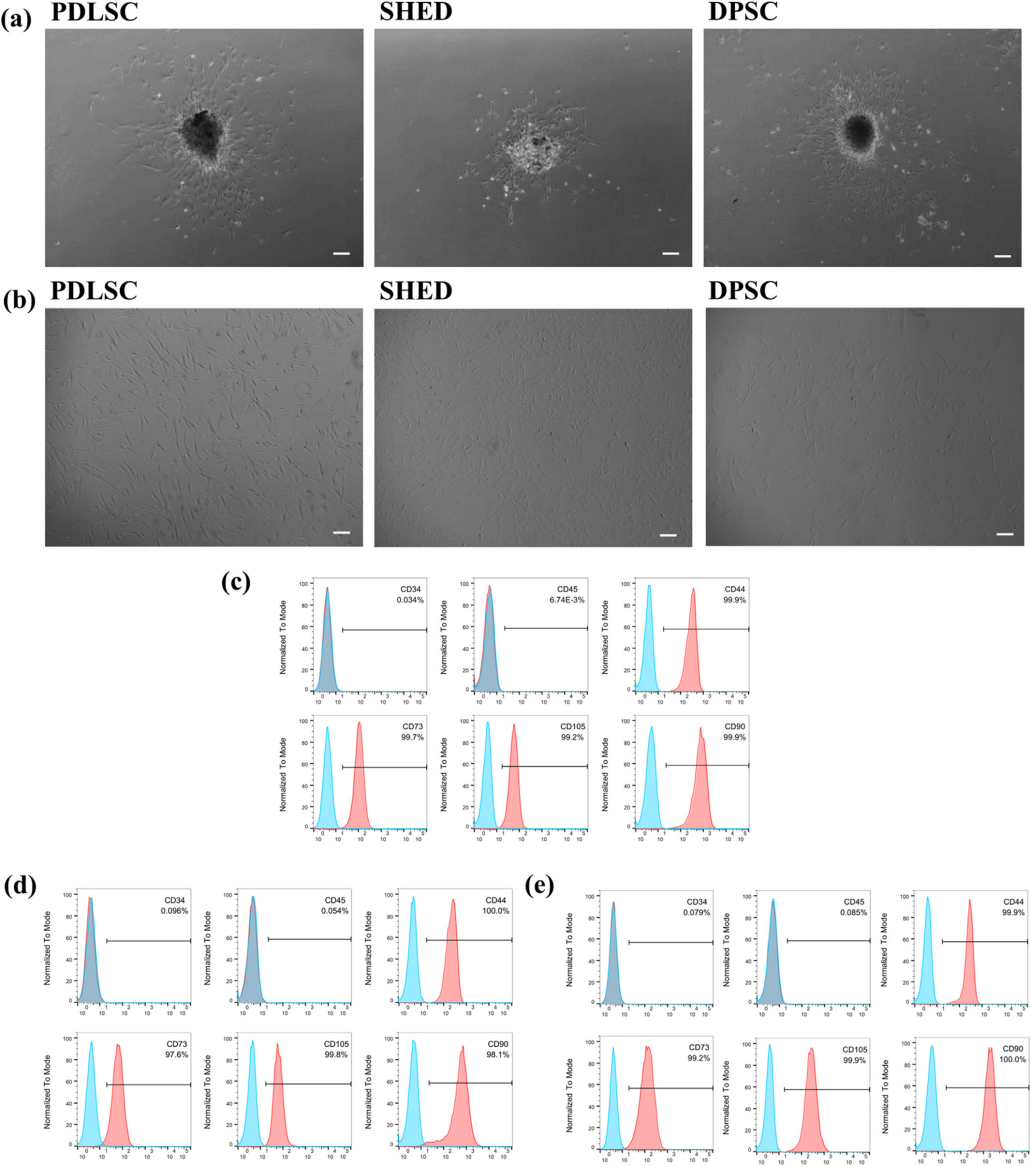
Characterization of SHEDs, DPSCs, and DPLSCs. (a) Primary stem cells. (b) Stem cells at P3. (c) Flow cytometry analysis of DPLSCs. (d) Flow cytometry analysis of SHEDs. (e) Flow cytometry analysis of DPSCs. (Scale bar = 200 μm).

The major morphology of SHEDs, DPSCs, and DPLSCs was typically spindle-like or fibroblast-like (Fig. 1a and b). Flow cytometric analysis confirmed that primary SHEDs, DPSCs, and DPLSCs all expressed high levels of the MSC marker CD44, CD73, CD90, CD105 and low levels of the hematopoietic cell marker CD34 and CD45 (Fig. 1 c, d, e). The results demonstrated that all cells cultured were mesenchymal stem cells and therefore possessed osteogenic potential.

### 3.2 Characterization of SHED-sEV and DPSC-sEV

**Fig. 2:**
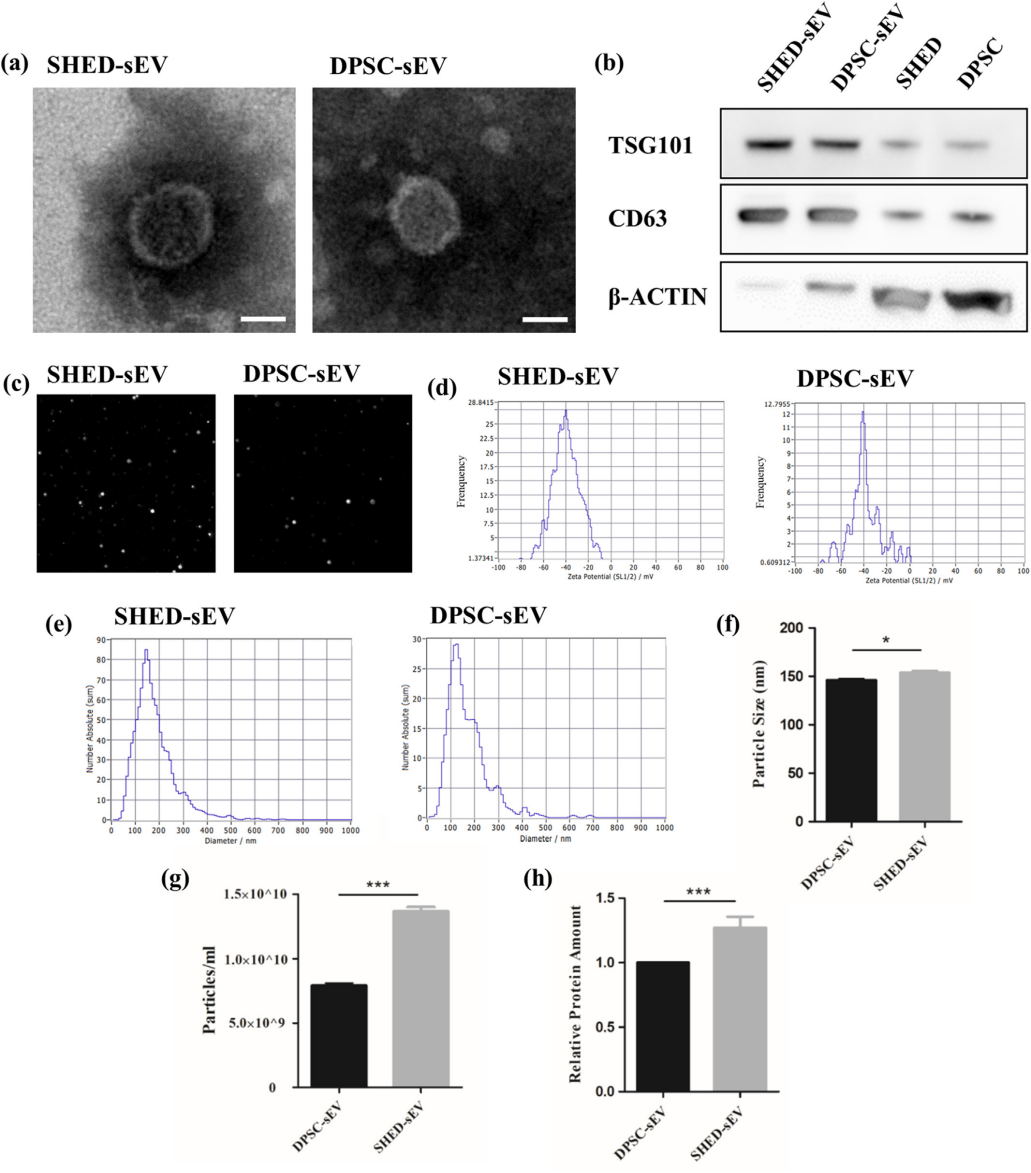
Characterization of SHED-sEV and DPSC-sEV. (a) The ultrastructures of SHED-sEV and DPSC-sEV were observed with a transmission electron microscope (TEM). (b) The expressions of the sEV-associated protein markers CD63 and TSG101 in purified SHED-sEV and DPSC-sEV were determined by western blotting. (c) SHED-sEV and DPSC-sEV tracked by NTA. (d) The zeta potential of SHED-sEV and DPSC-sEV determined by NTA. (e) The size distribution of SHED-sEV and DPSC-sEV determined by NTA. (f) The median particle sizes of DPSC-sEV and SHED-sEV determined by NTA (n = 3). (g) The concentrations of DPSC-sEV and SHED-sEV determined by NTA (n = 3). (h) The protein concentrations of DPSC-sEV and SHED-sEV determined by a Micro BCA assay (n = 3). (∗*p* < 0.05, ∗∗*p* < 0.01, ∗∗∗*p* < 0.001, Scale bar = 100 nm).

Using TEM, we observed that both sEVs exhibited classic cup-shaped morphology and had a bilayer membrane structure (Fig. 2a). Western blot analysis demonstrated that the two types of purified sEV expressed the sEV-associated protein markers (CD63, TSG101) (Fig. 2b). Zeta potentials of both sEVs determined by NTA demonstrated their stability (Fig. 2d). NTA also showed the size distribution of SHED-sEV and DPSC-sEV, the median diameters of SHED-sEV and DPSC-sEV were 154 nm and 146 nm, respectively (Fig. 2e and f). Moreover, we found that under the same conditions, the secretion volume of sEV from primary teeth was more than ten times that of permanent teeth (Fig. 2g). In addition, the protein concentration of SHED-sEV was also significantly higher than that of DPSC-sEV when other factors, including the supernatant volume and resuspension volume, were the same (Fig. 2h).

### 3.3 Endocytosis of SHED-sEV and DPSC-sEV by PDLSCs

**Fig. 3:**
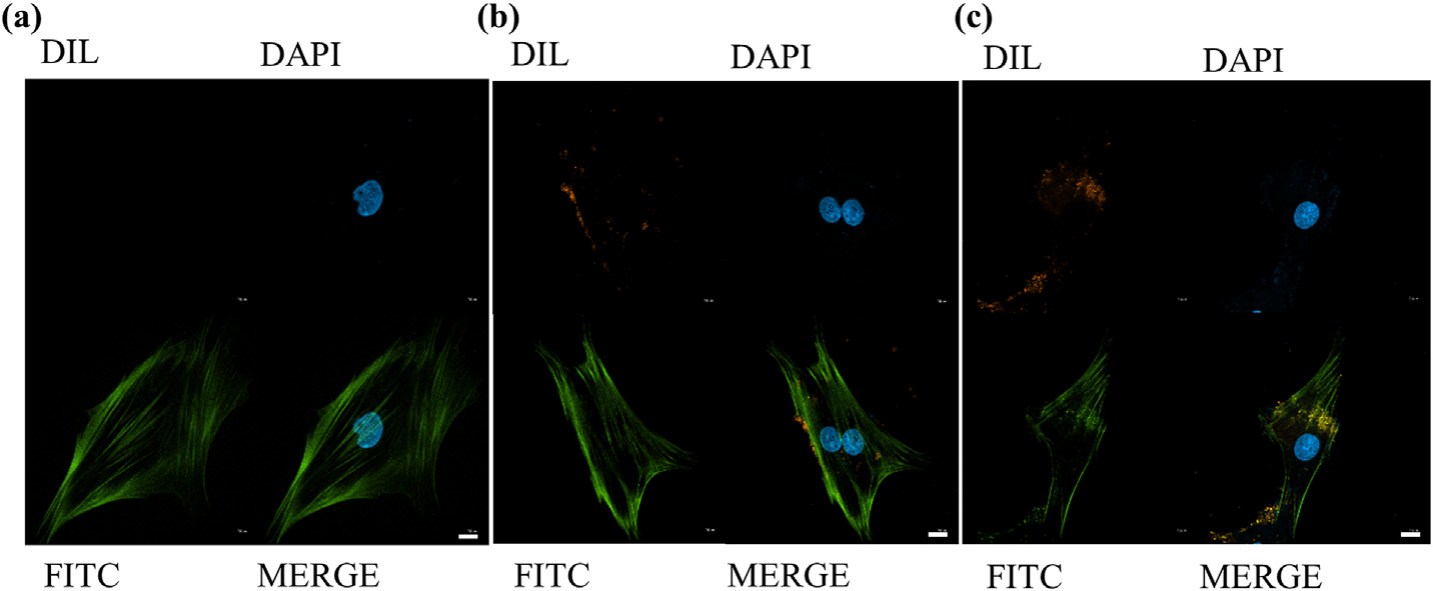
Endocytosis of sEV by PDLSCs. (a) Fluorescence images of DPLSCs cocultured without sEV for 24h. (b) Fluorescence images of DPLSCs cocultured with SHED-sEV for 24h. (c) Fluorescence images of DPLSCs cocultured with DPSC-sEV for 24h. (Scale bar = 10 μm).

Immunofluorescence staining shows that labeled SHED-sEV and DPSC-sEV emitted red fluorescence under 551 nm of excitation wavelength and located in the cytoplasm, which cannot be observed in the control group (Fig. 3). This phenomenon indicated that sEVs were endocytosed by DPLSCs: SEVs can penetrate the cell membrane and exert a certain effect.

### 3.4 SHED-sEV and DPSC-sEV promote the proliferation and migration of PDLSCs

**Fig. 4:**
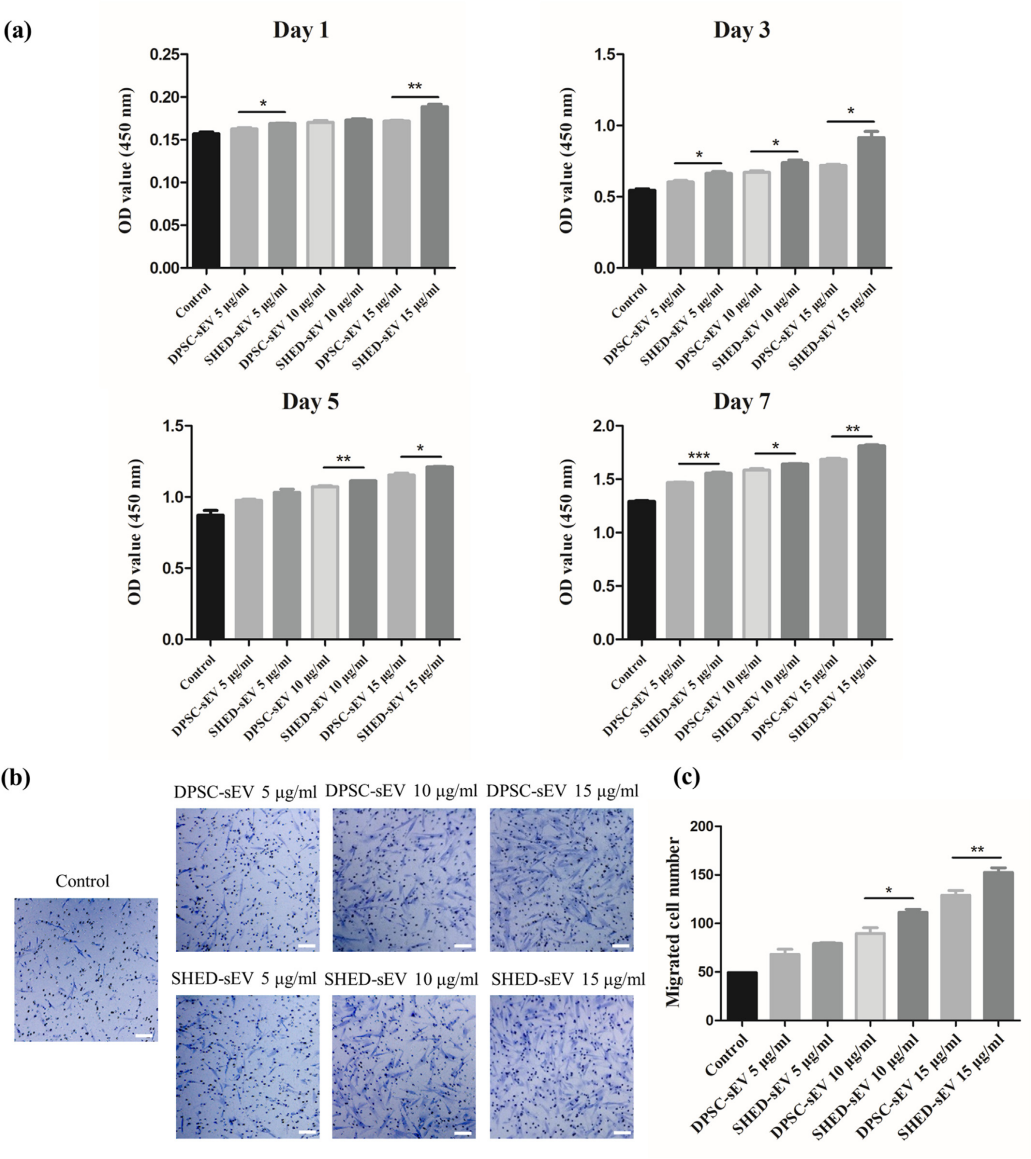
SHED-sEV and DPSC-sEV promote the proliferation and migration of PDLSCs. (a) The proliferation of DPLSCs incubated with different concentrations (0, 5, 10 and 15 μg/ml) of SHED-sEV or DPSC-sEV after 1, 3, 5, 7 days. (b) Representative image of migrating DPLSCs treated with different concentrations (0, 5, 10 and 15 μg/ml) of SHED-sEV or DPSC-sEV in the transwell assay ( × 10). (d) Quantification of the migrated cells in the transwell assay. (∗p < 0.05, ∗∗p < 0.01, ∗∗∗p < 0.001, Scale bar = 200 μm).

The proliferation ability of DPLSCs cocultured with different concentrations of sEVs was measured by CCK-8 assay. DPLSCs were cocultured with a medium, which included different concentrations (0, 5, 10 and 15 μg/ml) of SHED-sEV or DPSC-sEV for 1, 3, 5, and 7 days. According to the result (Fig. 4a), cells in 15 μg/ml sEV group proliferated faster compared with 5 μg/ml or 10 μg/ml sEV group. In addition, under the same concentration, cells in SHED-sEV group proliferated faster than those in DPSC-sEV group.

To detect the effects of sEV on the migration of PDLSCs, transwell assay was conducted. The transwell assay results showed that PDLSCs exerted a significantly higher migratory potency after being treated with 15 μg/ml sEV, compared with 5 μg/ml group or 10 μg/ml group (Fig. 4b and c). Additionally, compared with DPSC-sEV, SHED-sEV did better in promoting the migration of PDLSCs. Thus, 15 μg/ml was chosen as the effective concentration for succeeding experimentations.

### 3.5 SHED-sEV and DPSC-sEV promote the osteogenic differentiation of PDLSCs

**Fig. 5:**
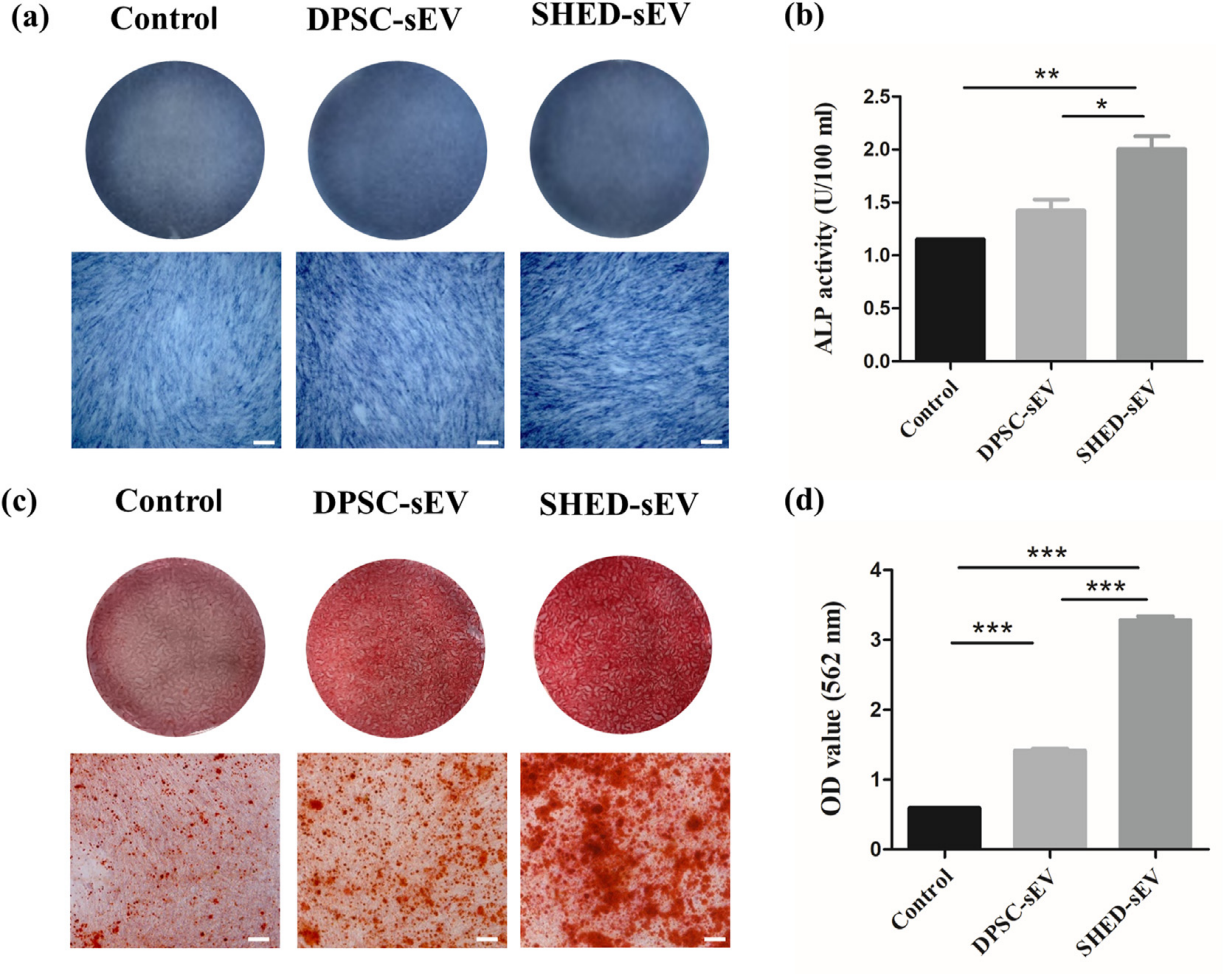
PDLSCs were cultured in presence or absence of 15 μg/ml SHED-sEV or DPSC-sEV. (a) Gross appearance and microscopic images of Alkaline phosphatase staining. (b) Quantitative detection of ALP activity for 7 days of coculture. (c) Gross appearance and microscopic images of Alizarin red staining. (d) Semi-quantitative interpretation of Alizarin red staining. (∗*p* < 0.05, ∗∗*p* < 0.01, ∗∗∗*p* < 0.001, Scale bar = 200 μm).

To evaluate whether SHED-sEV and DPSC-sEV can promote PDLSCs osteogenesis, PDLSCs were cultured with or without 15 μg/ml SHED-sEV or DPSC-sEV. The results of the ALP staining suggested that after 7 days of coculture, DPLSCs treated with SHED-sEV or DPSC-sEV showed higher ALP activity compared with the control group (Fig. 5a). And the ALP activity was higher in the SHED-sEV group with significant difference. The results of ALP assay after 7 days of coculturing was consistent with the appearance of the ALP staining (Fig. 5b), which indicated that sEV could play a promoting role in the early stage of osteogenic differentiation.

The results of Alizarin red staining after 14 days of coculturing are shown in Fig. 5c and d. Obviously, there was calcium nodule formation in the SHED-sEV and DPSC-sEV groups with a higher level of mineralization in the SHED-sEV group. The mineral deposition quantification further confirmed that there were more calcium deposits in SHED-sEV group than DPSC-sEV group and control group with significant difference.

**Fig. 6:**
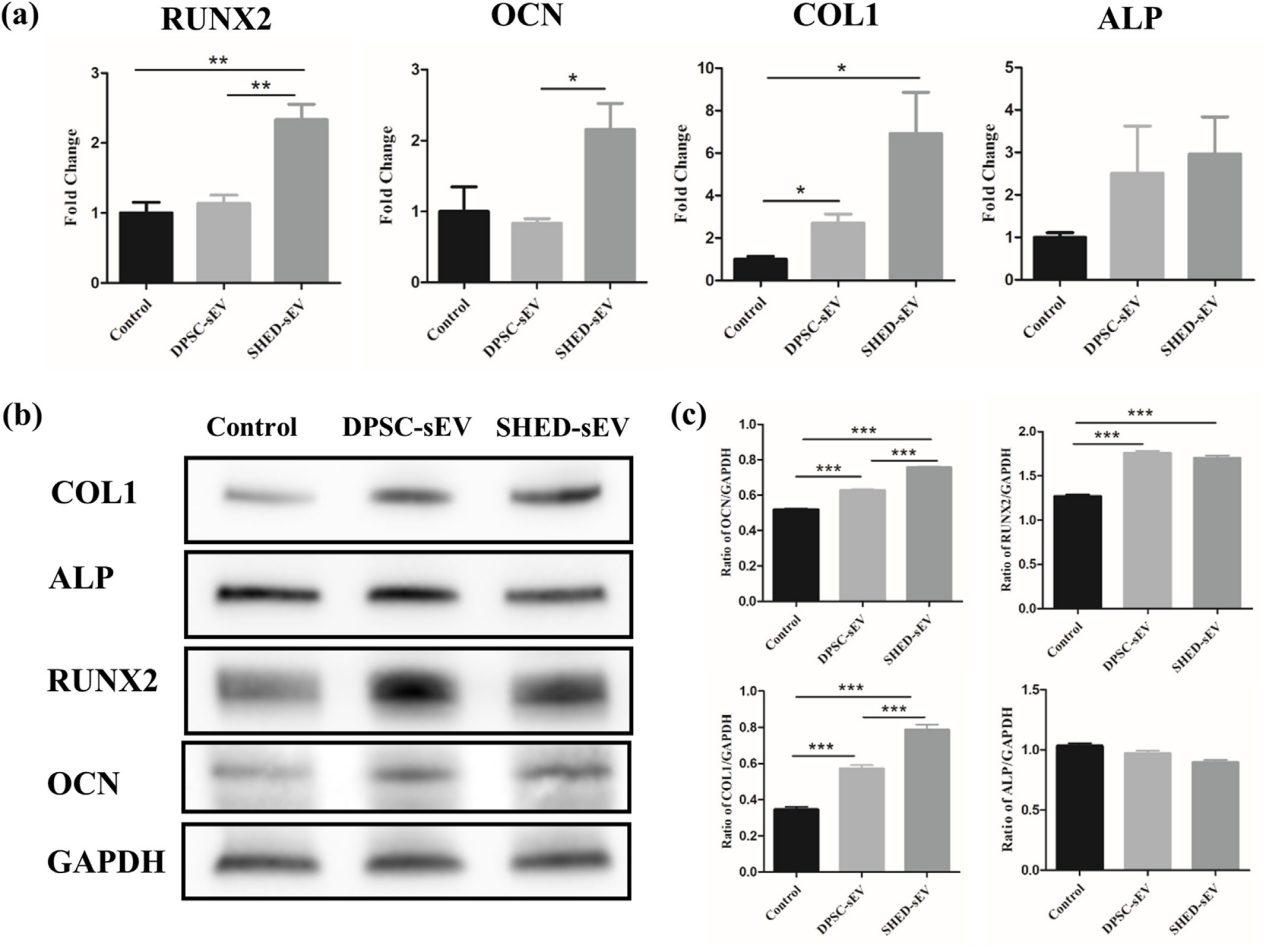
qRT-PCR and western blotting analyses of the expression levels of osteogenic proteins and genes of DPLSCs cocultured with 15 μg/ml SHED-sEV or DPSC-sEV. (a) Expression levels of osteogenic genes of 14 days' coculture tested by qRT-PCR. (b) Expression levels of osteogenic proteins of 14 days' coculture analyzed by western Blotting. (c) Quantitative analyses of western blotting. (∗*p* < 0.05, ∗∗*p* < 0.01, ∗∗∗*p* < 0.001).

To assess the effects of sEV on mRNA level of crucial osteogenesis-related genes, expressions of RUNX2、OCN、ALP and COL1 were measured by qPCR. After 14 days of osteogenic induction, RUNX2、OCN、ALP and COL1 were both upregulated in the SHED-sEV group and DPSC-sEV group. The expression of RUNX2 and COL1 increased significantly in SHED-sEV group. The expression levels of RUNX2、OCN、ALP and COL1 were higher in SHED-sEV group than in DPSC-sEV group and the differences were significant with respect to RUNX2 and OCN (Fig. 6a).

Furthermore, the protein expressions of RUNX2、OCN and COL1 were enhanced significantly by sEV (Fig. 6b and c). The expression levels of OCN and COL1 were higher in SHED-sEV group with significant difference. The expression levels of RUNX2 and ALP were higher in DPSC-sEV group without significant difference. Therefore, we established that SHED-sEV and DPSC-sEV can significantly encourage the osteogenic differentiation of PDLSCs. SHED-sEV is more potent than DPSC-sEV in this respect.

## 4 Dicussion

This in vitro study investigated the productivity of SHED-sEV and DPSC-sEV and their role in regulating PDLSCs osteogenic differentiation. Periodontal ligament cells belong to a heterogeneous group of cells. In addition to fibroblasts, which account for the most part of the group, there are also some cells expressing mesenchymal stem cell markers (such as Stro-1 and CD146). They have multidirectional differentiation potential and can differentiate into osteoblasts and cementoblasts [25]. Numerous studies have shown that PDLSCs play an important role in the repair and regeneration of periodontal tissue. Seo et al. used hydroxyapatite scaffolds for the first time to transplant human PDLSCs into artificial periodontal defects in the mandibular molars of immunodeficient mice. After 8 weeks, cementum and PDL-like structures appeared at the defect sites [25]. Iwasaki et al. transplanted human PDLSCs into artificially formed bifurcated defects in immunodeficient mice, with a probing depth of around 2 mm. Four weeks later, the defects in the control group were mainly filled with fibroblasts, collagen fibers and blood vessels, while in the transplantation group, new bone and cementum were formed [26]. However, stem cell transplantation faces several challenges, including high expenses, inadequate cell source, immune rejection and other safety problems, which greatly restrict its clinical application [27]. Recruiting endogenous PDLSCs by cell-free therapy based on paracrine secretion may provide a new way to repair periodontal bone defects.

Recently, basic research has indicated that the treatment effect of stem cell therapy is mainly contributed to the paracrine secretion of bioactive factors [28,29]. SEVs are important paracrine mediators. In recent years, sEVs secreted by dental stem cells have shown great potential in the field of periodontal tissue regeneration [21]. Dental mesenchymal stem cells originate from neural crest ectomesenchyme and exist in the stromal niches [30,31]. They express mesenchymal stem cell markers, and participate in regeneration of multiple tissues and immune regulation, both in vitro and in vivo [32,33]. These characteristics make these cells appropriate for therapeutic application, including dentin-pulp regeneration, periodontal regeneration and other diseases [34,35]. Since it's the paracrine secretion of bioactive factors that mediates intercellular communication, activates target cell signaling pathways, influences the biological behavior of cells in the microenvironment, and thereby induces the regeneration of defective tissue, a cell-free approach based on sEVs derived from dental mesenchymal stem cells has received widespread attention. Dental mesenchymal stem cells include dental pulp stem cells (DPSCs), stem cells from human exfoliated deciduous teeth (SHEDs), periodontal ligament stem cells (PDLSCs) and stem cells from apical papilla (SCAPs) [36]. In terms of tissue origin, DPSCs and SHEDs are more abundant than other dental cells, making it easier to obtain a large number of cells [30]. Numerous previous studies have investigated the therapeutic effect of SHED -sEV and DPSC-sEV in bone tissue engineering. Wang et al. found that SHED-sEV significantly promote the proliferation, migration and mineralization of PDLSCs by regulating BMP/Smad and Wnt/β-catenin signaling pathways [37,38]. Swanson's team reported that sEV derived from DPSCs can enhance osteogenic differentiation of BMSCs. A controlled release system of DPSC-sEV was successfully designed to implant DPSC-sEV into rat skull defects to accelerate bone healing in vivo [39]. However, the productivity and osteogenic-inducing ability of DPSC-sEV and SHED-sEV are not clear. Therefore, our study focused on sEVs derived from SHEDs and DPSCs and tries to find a better source of sEVs for periodontal bone regeneration.

In this study, we successfully isolated and cultured human PDLSCs, SHEDs, and DPSCs. Flow cytometry showed that PDLSCs, SHEDs, and DPSCs expressed extremely low levels of CD45 and CD34, while high levels of CD44, CD73, CD90, and CD105, consistent with the basic characteristics of mesenchymal stem cells. Highly purified SHED-sEV and DPSC-sEV were separated by ultracentrifugation. The secretion volume of sEV from primary teeth and permanent teeth were compared by nanoparticle tracking analysis. Under the same conditions, the secretion volume of sEV from primary teeth was more than ten times that of permanent teeth. The results of BCA quantification also showed that the protein concentration of SHED-sEV was higher than DPSC-sEV when other factors are the same. All the results support that the secretory efficiency of SHED-sEV is much higher than that of DPSC-sEV.

The proliferation, migration and osteogenic differentiation of PDLSCs are crucial to the regeneration of periodontal bone defects. The sEV-based cell-free therapy requires endogenous PDLSCs to migrate to the damaged site, and the stem cells must have self-renewal ability to ensure continuous tissue repair. Results of cck8 and transwell assay indicated that the proliferation and migration of PDLSCs were enhanced by both SHED-sEV and DPSC-sEV, especially SHED-sEV. Furthermore, PDLSCs treated with 15 μg/ml sEVs showed higher proliferation and migration ability compared with the 5 μg/ml and 10 μg/ml group, which indicated that the effect of sEV on the function of PDLSCs may be concentration-dependent. Alkaline phosphatase (ALP) and calcium nodules formation are the markers of mature osteoblasts. We used ALP staining, ALP assay and alizarin red staining to evaluate the osteoblasts-like cells derived from PDLSCs. Results showed that sEV could promote osteoblast differentiation. However, it requires confirmation whether more cells enhanced mineralization in the sEV group or sEV indeed upregulated the osteogenesis-related genes and proteins and then promoted mineralized matrix formation.

Subsequent experiments focused on osteogenesis-related genes and proteins (ALP, RUNX2, OCN, COL1). As we know, ALP increases inorganic phosphate local rates and facilitates mineralization and reduces the extracellular pyrophosphate concentration, an inhibitor of mineral formation [40]. RUNX2 is a key transcription factor regulating genes that encode for proteins involved in osteogenesis [41,42]. In addition, bone extracellular matrix proteins such as OCN and specific collagen such as COL1, which are secreted by osteoblasts, regulate metabolism and play a vital role in bone formation [43,44]. According to the results shown in Fig. 6a–c, both SHED-sEV and DPSC-sEV could increase the expression of osteogenesis-related genes and proteins (RUNX2, ALP, OCN, and COL1), and the expression level is higher in the SHED-sEV group. All these results demonstrate SHED-sEV as a potential bioactive component for improving periodontal bone regeneration.

Our study further confirmed that the biological functions of sEV generally corresponded to their parent cells. SHEDs showed better ability in proliferation, migration, and osteogenic differentiation than DPSCs. Similarly, SHED-sEV did better in promoting proliferation, migration, and osteogenic differentiation than DPSC-sEV. These results may provide reference for further research and treatment of periodontal bone defects. However, it is still unclear which specific components transferred to PDLSCs result in such changes. Subsequent studies will further compare the composition of SHED-sEV and DPSC-sEV, looking for the key components in sEV that regulate the function of PDLSCs, trying to elucidate the potential molecular mechanism.

In conclusion, we demonstrated that the secretory efficiency of SHED-sEV was much higher than that of DPSC-sEV. Both SHED-sEV and DPSC-sEV can promote the proliferation, migration, and osteogenic differentiation of PDLSCs. SHED-sEV showed advantages over DPSC-sEV, conforming to the properties of SHEDs and DPSCs. Thus SHED-sEV is a more promising candidate for periodontal bone regeneration.

## Declaration of competing interest

The authors declare they have no competing interests.
